# DYADS’ POSITIVE AND NEGATIVE AFFECT ASSOCIATION WITH MENTAL HEALTH AND PERCEIVED GENERAL HEALTH

**DOI:** 10.1093/geroni/igad104.2437

**Published:** 2023-12-21

**Authors:** Cristina de Rosa, Weijun Wang, Yu-Ping Chang

**Affiliations:** University at Buffalo, The State University of New York, Buffalo, New York, United States; University at Buffalo, The State University of New York, Buffalo, New York, United States; University at Buffalo, The State University of New York, Buffalo, New York, United States

## Abstract

Positive and negative affect may impact health in older adults and their family caregivers. However, how members of a caregiving dyad may influence each other’s health outcomes is not well understood. The purpose of this analysis was to simultaneously examine whether both dyad members’ positive and negative affect were associated with their own and each other’s mental health (depression and anxiety) and perceived general health. Dyadic data were obtained from the National Health and Aging Trends Study (NHATS) (2021) linked to the National Study of Caregiving (NSOC) IV (2021). The sample consisted of 1,209 pairs of adults aged 71 and older and their respective primary caregivers, defined as the family member or friend who provided the greatest number of hours of care in the preceding month. Actor-partner interdependence models were used to examine associations between positive and negative affect, mental health, and perceived general health, controlled for dyad members’ own gender, age, and race/ethnicity. Results indicated that positive and negative affect significantly predicted caregivers’ and older adults’ own mental health and perceived general health. Furthermore, older adults’ negative affect significantly predicted caregivers’ expressing little interest or pleasure in doing things (b = 0.033 (0.016), p = .046). Our results suggest that an individual’s mood influences their mental health and perceived general health even when considering partner effects. Caregivers’ interest or pleasure is particularly impacted by older adults’ negative affect. Dyadic supportive strategies to improve mood could be beneficial for both older adults and their family caregivers.

